# Takotsubo cardiomyopathy mimicking an apical hypertrophic cardiomyopathy

**DOI:** 10.1007/s10554-024-03109-8

**Published:** 2024-05-04

**Authors:** N. Maurizi, P. Antiochos, P. Monney

**Affiliations:** Service of Cardiology, University Hospital of Lausanne, Lausanne, Switzerland

**Keywords:** Hypertrophic cardiomyopathy, Takotsubo, Cardiac magnetic resonance

## Abstract

A 45-years old woman presented for dyspnea and cardiac chest pain. ECG showed deep T-wave inversion while CMR showed normal ejection fraction, hypertrophy and systolic obliteration of the apex suggesting apical HCM. Myocardial oedema was noted at the apex. Complete regression of hypertrophy and myocardial edema was observed after 2 months, and a final diagnosis of subacute Takotsubo was made.

A 45 years old woman was admitted for NYHA class II-III exertional dyspnea associated with typical cardiac chest pain lasting for 1 week. She had no previous medical history apart from arterial hypertension treated with Lisinopril 10 mg od, and she was physically active. No family history of heart muscle disease or sudden cardiac death was present. Physical examination was unremarkable.

ECG was in sinus rhythm at 63 bpm, with a normal atrioventricular and intraventricular conduction. There was no Q-waves or ST-segment changes but diffuse deep T wave inversion was present from V2 to V6, and in leads DI, DII, aVL and aVF. QTc (Bazett) interval was severely prolonged to 570 ms (Fig. 1). Initial routine blood tests including haemogram, renal and thyroid function tests were normal. A slight troponin increase was present (hsTnT 19 mcg/L, Normal < 14 mcg/l) together with a severe increase in NT-proBNP levels (6085 ng/l, Normal < 125 ng/l). Echocardiography showed a non-dilated left ventricle with localized left ventricular hypertrophy of the apex and inferior septum (maximal wall thickness of 22 mm). LVEF was 72% with no regional wall motion abnormalities, and there was complete obliteration of the apex in systole. The left atrium was not dilated and the other parameters of diastolic function were within normal range (E/A ratio of 1; mean E/e’ ratio 7,5). No valve disease was noted. (Fig. 2, panel A,B). Coronary angiogram showed unobstructed coronary arteries. Cardiac magnetic resonance performed at the 4th hospital day also showed non-dilated hypercontractile LV with a LVEF of 74% and no regional wall motion abnormalities. Maximal LV wall thickness was 17 mm at the inferior apical segment and wall thickness was normal in the basal segments. Using T2-mapping sequences, the T2-relaxation time of the myocardium was increased in the mid to apical segments (between 58 and 63 ms) but remained normal in the basal segments (between 45 and 51 ms). There was no late gadolinium enhancement (LGE). (Fig. 1, panel C, D, E, F). Additional blood tests performed in the work-up of cardiac infiltration of myocarditis were normal, including serum and urine electrophoresis was within limits, serology for Lyme disease, HIV, HAV, HBV, and HCV, anti-nuclear antibody and rheumatoid factor.

**Fig. 1:**
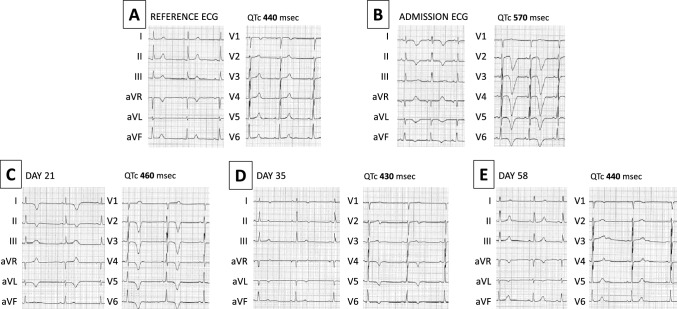
Electrocardiographic evolution during follow-up. On Panel A, the reference ECG with the QTc interval is displayed, whereas on Pane B, the abnormal ECG at the admission is showed with a QTc of 570 ms. The evolution at day 21 (Panel C), day 35 (Panel D), day 58 (Panel E) are presented

**Fig. 2:**
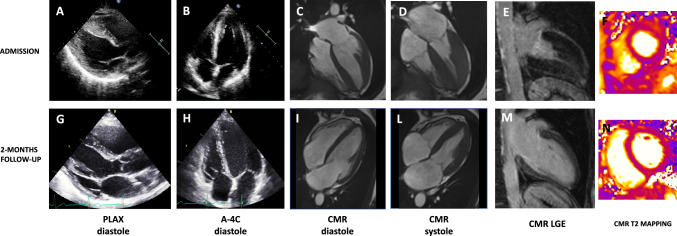
Echocardiographic and cardiac magnetic resonance presentation and evolution at two month. On Panel A and B the echocardiographic parasternal long axis and apical 4 chamber views at the admission are presented. In panel C, D, E and F CMR results are shown, particularly diastolic apical 4 chamber view (C), systolic apical 4 chamber view (D), 2 chamber LGE contrast imaging (E) and T2 contrast mapping at the medioventricular level showing high relaxation times (F). On the bottom, evolution at 2 months is represented. In panel G and H, the follow-up the parasternal long axis and apical 4 chamber views are presented, showing regression of the ‘pseudo-apical hypertrophy’. In panel I, L, M, N CMR results are shown, particularly diastolic apical 4 chamber view (L), systolic apical 4 chamber view (M), the 2-chamber LGE contrast imaging and T2 contrast mapping at the medio-ventricular to basal level with normalized T2 values (N)

The combination of apical hypertrophy with systolic apical obliteration and deep T-wave inversion in the front ECG leads is typically associated with the apical form of hypertrophic cardiomyopathy (apHCM). However the clinical picture was not completely characteristic for apHCM, as myocardial oedema and severe QTc prolongation are usually not associated with this diagnosis. Considering the relatively acute presentation of the symptoms, the mild increase in hsTnT together with a severe increase in NT-proBNP, we considered a differential diagnosis of Takotsubo cardiomyopathy at a subacute stage (i.e. after recovery of the apical hypokinesia and ballooning). Other less likely diagnosis included myocarditis and coronary artery spasm.

Cardiac monitoring did not reveal any significant arrhythmia and the symptoms resolved after few days under low-dose betablocker treatment (metoprolol 25 mg od). The patient was discharged after 8 days and the discharge ECG showed partial normalization of T-waves inversion and the QTc interval improved to 489 ms.

After 2 months, the patient remained asymptomatic with no clinical signs of heart failure. Cardiac troponin T and NT-pro BNP levels were 8 ng/L and 85 pg/L, respectively. ECG was in sinus bradycardia (45 bpm) and the profound changes observed at admission almost completely resolved: the pathological T-wave inversion was no more observed and the QTc interval was normal (440 ms) (Fig. 1, Panel E). Both echocardiography and CMR showed a non-dilated left ventricle with a normal LVEF, and disappearance of apical hypertrophy and systolic obliteration. (Fig. 1, panel G, H). On CMR, No LGE was observed, and T2 relaxation times were normal in all myocardial segments (relaxation times 45–50 ms) indicating regression of the apical myocardial oedema. Considering this remarkable evolution, we concluded that the apical wall thickening initially detected was only related to severe myocardial oedema but not to myocardial hypertrophy. HCM could be confidently excluded, as hypertrophy is not reversible in this situation. We made a final diagnosis of Tako-tsubo cardiomyopathy at a subacute stage, a stage, where systolic function already recovered but the apical myocardium was still severely oedematous. The typical ECG evolution characterized by transient profound T-wave inversion and QT prolongation also supported this diagnosis.

This case illustrates several important points: (i) Takotsubo cardiomyopathy can mimic apical hypertrophic cardiomyopathy (one case previously described in the literature) at the specific time point where apical ballooning and akinesia already resolved but strong myocardial oedema is still present. However, this is a transient process and, upon resolution of the myocardial oedema, the wall thickness returns to normal. (ii) Myocardial wall thickening should be interpreted with caution in the presence of increased myocardial T2 relaxation time by CMR, as myocardial oedema is typically not a feature of hypertrophic cardiomyopathy. (iii) Transient severe QTc prolongation and diffuse T-wave inversion are typically observed with Takotsubo cardiomyopathy, while apical HCM usually causes profound T-wave inversion in the front ECG leads with no significant QT prolongation (Fig. 1). A comprehensive evaluation including history, ECG, precise morphological assessment of the LV and myocardial tissue characterization with CMR is of utmost importance in case of suspected apical HCM [1, 2].

## Data Availability

Anonymized data would be available upon acceptable request.
